# Estrogen-Induced Nongenomic Calcium Signaling Inhibits Lipopolysaccharide-Stimulated Tumor Necrosis Factor α Production in Macrophages

**DOI:** 10.1371/journal.pone.0083072

**Published:** 2013-12-23

**Authors:** Limin Liu, Ying Zhao, Keming Xie, Xiaodong Sun, Yuzhen Gao, Zufeng Wang

**Affiliations:** 1 Department of Pathology and Pathophysiology, Medical College of Soochow University, Suzhou, Jiangsu, China; 2 Department of Forensic Medicine, Medical College of Soochow University, Suzhou, Jiangsu, China; University of Illinois at Chicago, United States of America

## Abstract

Estrogen is traditionally thought to exert genomic actions through members of the nuclear receptor family. Here, we investigated the rapid nongenomic effects of 17β-estradiol (E_2_) on tumor necrosis factor α (TNF-α) production following lipopolysaccharide (LPS) stimulation in mouse bone marrow-derived macrophages (BMMs). We found that LPS induced TNF-α production in BMMs via phosphorylation of p38 mitogen-activated protein kinase (MAPK). E_2_ itself did not affect the MAPK pathway, although it attenuated LPS-induced TNF-α production through suppression of p38 MAPK activation. Recently, G protein-coupled receptor 30 (GPR30) was suggested to be a membrane estrogen receptor (mER) that can mediate nongenomic estradiol signaling. We found that BMMs expressed both intracellular estrogen receptors (iER) and mER GPR30. The specific GPR30 antagonist G-15 significantly blocked effects of estradiol on LPS-induced TNF-α production, whereas an iER antagonist did not. Moreover, E_2_ induced a rapid rise in intracellular free Ca^2+^ that was due to the influx of extracellular Ca^2+^ and was not inhibited by an iER antagonist or silencing of iER. Ca^2+^ influx was also induced by an impermeable E_2_ conjugated to BSA (E_2_-BSA), which has been used to investigate the nongenomic effects of estrogen. Consequently, Ca^2+^, a pivotal factor in E_2_-stimulated nongenomic action, was identified as the key mediator. The inhibitory effects of E_2_ on LPS-induced TNF-α production and p38 MAPK phosphorylation were dependent on E_2_-triggered Ca^2+^ influx because BAPTA, an intracellular Ca^2+^ chelator, prevented these effects. Taken together, these data indicate that E_2_ can down-regulate LPS-induced TNF-α production via blockade of p38 MAPK phosphorylation through the mER-mediated nongenomic Ca^2+^ signaling pathway in BMMs.

## Introduction

In addition to its pivotal role in sexual development and reproduction, the sexual steroid hormone estrogen has been reported to regulate numerous immune and inflammatory responses, especially during autoimmune and infectious pathophysiological processes [1]–[3]. These actions of estrogen are thought to mainly result from its specific effects on the different cellular components of the immune system because most, if not all, of these components have been demonstrated to express estrogen receptors [4]–[6]. Macrophages are important in the immune-modulatory role of estrogen [4]. There is a wealth of clinical and laboratory data demonstrating that sex hormones affect the immune system by modulating the function of the monocyte-macrophage system by mechanisms that include macrophage activation and synthesis of cytokines [7], [8]. The control of the production of macrophage cytokines can greatly facilitate the treatment of many immunoinflammatory diseases such as septic shock, rheumatoid arthritis, cerebral malaria, and autoimmune diabetes [9], [10].

Macrophages exhibit a particularly vigorous response to lipopolysaccharide (LPS), which is a potent activator of the immune system that induces a variety of inflammatory modulators such as tumor necrosis factor α (TNF-α), nitric oxide, interleukin-1, interleukin-6, and prostaglandins [11]. TNF-α is a pluripotent cytokine that is produced predominantly by activated macrophages and has multiple biologic effects including cell differentiation, proliferation, and multiple pro-inflammatory effects. Deregulated TNF-α production has been correlated with numerous autoimmune disorders, including rheumatoid arthritis and systemic lupus erythematosus [12], [13]. In response to LPS, the mitogen-activated protein kinase (MAPK) cascades are activated in macrophage [14], [15]. MAPKs are signaling molecules that play important roles in the regulation of immune responses including cell activation and cytokine production. There are three major MAPK dependent pathways: p38 MAPK, extracellular-regulated protein kinase (ERK) 1/2, and c-Jun NH_2_-terminal kinase (JNK). The phosphorylated MAPKs transduce their signals downstream and promote activation and translocation of transcription factors that subsequently regulate the expression of different cytokine genes and the biological functions of cells [16]–[18].

In recent years, the investigation of estrogen-induced signaling pathways in LPS-activated macrophages has been important and necessary for discovering potential therapeutic targets and drug for immunoinflammatory diseases. The main endogenous estrogen, 17β-estradiol (E_2_), has traditionally been thought to mediate its effects via intracellular estrogen receptors (iER) that are located in the cytoplasm or on the nuclear membrane; thus, studies have investigated the effect of E_2_ on transcription factors in the regulation of target genes [19], [20]. However, recent findings indicate that E_2_ also acts on the plasma membrane to initiate signaling pathways in the cytoplasm and regulate cellular functions, and these pathways are referred to as nongenomic. These nongenomic effects of E_2_ that are mediated by membrane estrogen receptors (mER), or perhaps other ligands, can induce the generation of the second messengers Ca^2+^ and nitric oxide and activate several signaling pathways [21]–[24]. Primary macrophages have been shown to express G protein-coupled receptor 30 (GPR30), which may function as a novel transmembrane estrogen receptor and can mediate rapid nongenomic events. Estrogen may utilize this non-classical estrogen receptor to limit potentially lethal inflammatory responses. [25]

Although E_2_ produces salutary effects on macrophage activation and the synthesis of cytokines, the precise molecular mechanisms of these effects are still unknown. In the present study, we examined the effect of E_2_ on TNF-α production and explored the molecular mechanism of this effect in LPS-stimulated mouse bone marrow-derived macrophages (BMMs). Our data indicate that E_2_ can down-regulate LPS-induced TNF-α production via blockade of p38 MAPK phosphorylation through the mER-mediated nongenomic Ca^2+^ signaling pathway in BMMs. Investigation of the mechanism by which estrogen induces the synthesis of cytokines in macrophages is important and necessary for discovering potential therapeutic targets and treating various immunoinflammatory diseases.

## Materials and Methods

### Ethics Statement

All animal work was conducted in adherence to the guidelines of the Chinese Ministry of Science and the Technique for Accreditation of Laboratory Animal Care and was approved by the Institutional Animal Care and Use Committee of Soochow University.

### Reagents

RPMI 1640 and FBS were purchased from HyClone (Logan, UT, USA). LPS, E_2_, E_2_ conjugated to BSA (E_2_-BSA), E_2_-BSA-FITC, BAPTA, tamoxifen, ICI 182780, Fura-2/acetoxymethylester and other chemical reagents were purchased from Sigma (St. Louis, MO, USA). Anti-ERα, anti-ERβ, anti-GPR30, and HRP and FITC-conjugated secondary antibodies were purchased from Santa Cruz Biotechnology (Santa Cruz, CA, USA). The specific GPR30 inhibitor G-15 was purchased from Millipore (Darmstadt, Germany). SB203580, PD98059, SP600125, the antibodies against p38, phospho-p38, ERK 1/2, phospho-ERK 1/2, JNK, and phospho-JNK were purchased from Cell Signaling Technology (MA, USA). F4/80 antibody-FITC was from Serotec (Oxford, UK). Before treatment, E_2_ and E_2_-BSA were tested for LPS contamination using a Limulus amebocyte lysate assay (Biowhittaker, Walkersville, MD), and the level of endotoxin was found to be insignificant.

### Mice

C57BL/6j mice were obtained from Shanghai Experimental Animal Center of the Chinese Academy (Shanghai, P. R. China). Mice received a standard diet and water ad libitum, and the protocol was fully approved by the Chinese Ministry of Science and was in accordance with the Technique for Accreditation of Laboratory Animal Care. Mice were sacrificed by cervical dislocation.

### Preparation and culture of BMMs

Murine bone marrow cells were obtained from three-week old male C57BL/6j mice by flushing the femurs as previously described [26]. Bone marrow cells were grown in RPMI 1640 complete medium supplemented with 15% (v/v) L929 cell-conditioned medium as a source of macrophage- and monocyte-colony stimulating factor in 37°C and 5% CO_2_ under saturated humidity conditions. On day 5, bone marrow cells were harvested by scraping. The FITC-conjugated antibody F4/80 was used as a membrane surface marker that is specific for macrophages and was added at 10 µg/10^6^ cells. After incubation for 30 min on ice in the dark, the cells were washed again and resuspended in PBS. F4/80-positive bone marrow cells (BMMs) were sorted with a FACSCalibur (Becton Dickinson, USA) flow cytometer and replated in 6-well dishes at 37°C, 5% CO_2_, and 96% humidity for further culturing. The cells were cultured in phenol red-free RPMI 1640 containing 10% charcoal-dextran FBS for 48 h before treatment.

### Real-time PCR

Total RNA was prepared using Trizol reagent (Invitrogen) according to the manufacturer's instruction. The mRNA expression of ER was analyzed by real-time PCR performed with an Applied Biosystems 7300 Real-Time PCR system using SYBR Green PCR Core Reagents (Applied Biosystems). The primers used for ERα were as follows: GCCGAGGAGGGAGAATGTTG (sence) and CGCCAGACGAGACCAATCAT (antisence). The primers used for ERβ were as follows: CATCAGTAACAAGGGCATGG (sence) and CACTGAGACTGTAGGTTCTG (antisence). The samples were amplified with a two-step reaction at 95°C for 15 s and 64°C for 1 min for 40 cycles. Relative quantitative evaluation of target gene levels was performed by comparing ΔCt, where Ct is the threshold concentration. Product accumulation was measured during the extension phase, and all samples were run in triplicate.

### ER silencing

Cells were transfected with 50 nM ERα siRNA, ERβ siRNA, or control siRNA (Invitrogen, CA, USA) in Lipofectamine RNAimax (Invitrogen, CA, USA) according to the manufacturer's instructions. After 72 h of transfection, the cells were switched to phenol red-free RPMI 1640 supplemented with 10% charcoal-stripped FBS for 48 h before being harvested.

### Preparation of subcellular fractions

Cells were suspended in HEPES buffer followed by sonication for 10 sec on a sonicator. The homogenate was centrifuged at 1000× *g* for 7 min to pellet the nuclear material, and the resulting supernatant was centrifuged at 20, 000× *g* for 20 min to pellet the membrane fraction. Cytoplasmic fractions were obtained by centrifugation at 100,000× g for 1 h of the remaining supernatant after the 20,000× g spin. Subcellular fractions were stored at −80°C for up to 2 d before analysis.

### Western blotting

Aliquots containing 30 µg of protein were subjected 10% SDS-PAGE, followed by electrotransfer to PVDF membranes (Millipore, MA, USA). Blots were probed with primary antibody for 2 h at a dilution of 1∶1000, followed by incubation with HRP-conjugated secondary antibody for 1 h at a dilution of 1∶10000. Detection was performed using the enhanced chemiluminescence system (Biocolors, Shanghai, PR China). Images were scanned, and signal density was quantified using the ChemiImager 5500 imaging software (Alpha Innotech, San Leandro, CA, USA).

### Flow cytometry

Both intact and permeabilized cells (prefixed with 0.5% paraformaldehyde) were incubated with anti-ERα or anti-ERβ antibody (1∶150, 30 min) followed by FITC-conjugated secondary antibody (1∶320, 30 min). Nonpermeabilized cells were pretreated with E_2_-BSA-FITC (1×10^−6^ M) or with BSA-FITC alone as control for 30 min and then fixed with paraformaldehyde. Nonpermeabilized cells were pretreated with anti-GPR30 (1∶150, 30 min) followed by FITC-conjugated secondary antibody and then fixed with paraformaldehyde. Flurescence intensity was analyzed with a FACScan (Becton Dickinson, USA), and samples of 10,000 cells were gated on the basis of forward and side scatter. The data were evaluated using Cellquest software according to the manufacturer's instructions.

### Confocal laser scanning microscopy (CLSM)

Cells were allowed to adhere onto coverslips overnight and then incubated with anti-ER antibody, anti-GPR30 antibody, or E_2_-BSA-FITC as described above. The coverslips were mounted onto slides and analyzed with LEICA TCS NT CLSM version 1.5.451 (Leica Lasertechnik, Heidelberg, Germany) with FITC fluorescence excitation at 488 nm.

### Measurement of intracellular Ca^2+^

Cells were incubated with 3 µM Fura-2/acetoxymethylester for 35 min in HEPES buffer. Intracellular Ca^2+^ was detected using a Hitachi F-4500 spectrofluorometer with Fura-2 fluorescence excitation at 340/380 nm and emission at 510 nm. Intracellular free Ca^2+^ concentration ([Ca^2+^]_i_) were computed from the ratio of 340 to 380 nm fluorescence values using the equation as described previously [27].

### ELISA analyses

The TNF-α protein levels in the BMM culture supernatants were determined using an ELISA kit from R&D Systems (Minneapolis, MN, USA), according to the manufacturer's instructions.

### Statistics

The results are expressed as the means ± the standard errors of the mean (SEM),and the obtained data were evaluated with one-way ANOVA as appropriate. Statistical difference was accepted at P<0.05.

## Results

### Effect of p38 MAPK on LPS-induced TNF-α production in BMMs

**Figure 1:**
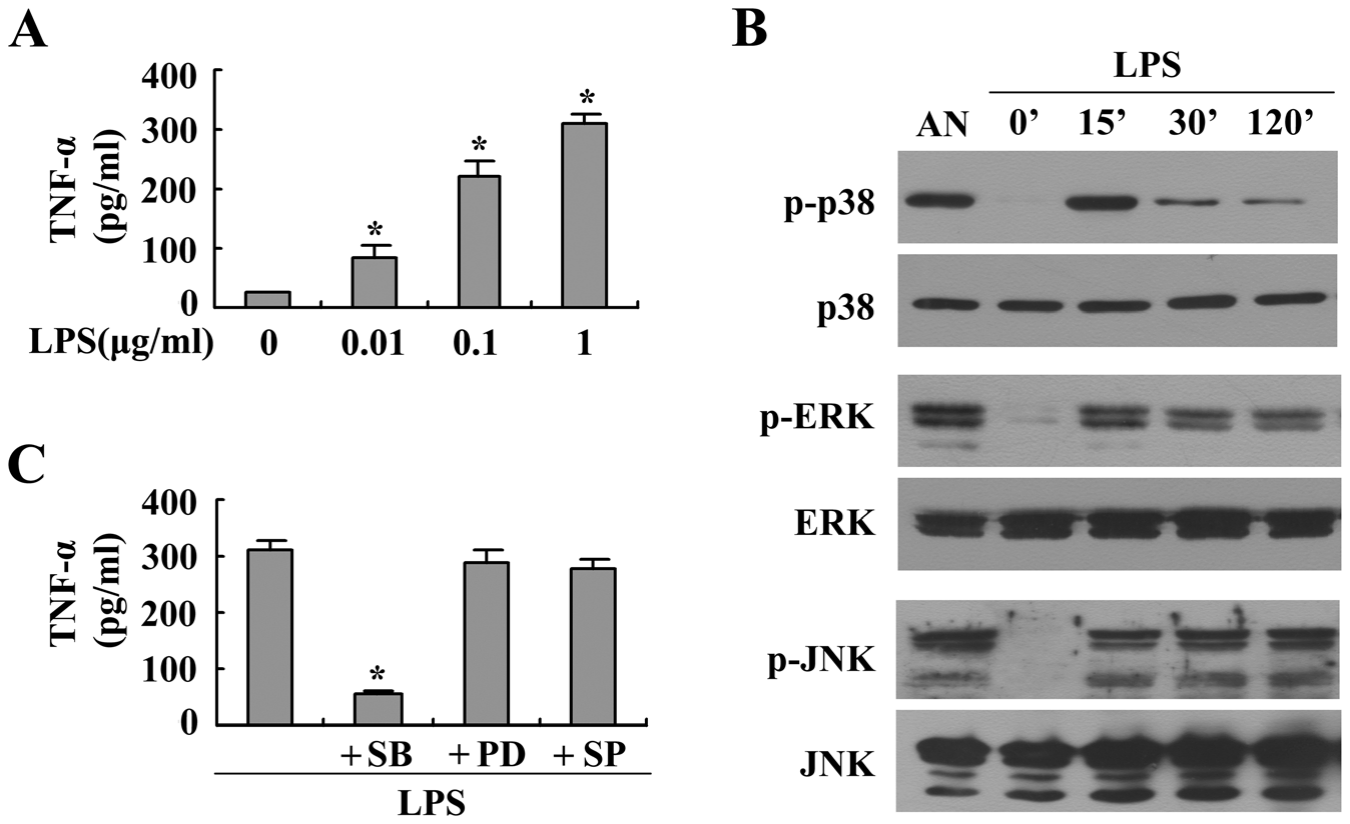
Effect of p38 MAPK on LPS-induced TNF-α production in BMMs. (A) Cells were treated with different concentrations of LPS for 24 h. Secretion of TNF-α in culture supernatants was detected using ELISA. The results are expressed as the means ± the SEMs of three independent experiments. (B) The cells were treated with LPS (1 µg/ml) for different periods. Protein extracts were subjected to western blotting to detect the phosphorylated and total forms of three MAPK molecules, p38 MAPK, ERK1/2 and JNK, using anti-MAPK antibody. Stimulation with anisomycin (AN, 10 µg/ml for 30 min) was used as a positive control. Representative blots are shown, and the results were verified by at least three independent experiments. (C) Cells were pretreated with specific inhibitors of p38 MAPK (SB203580, 20 µM), ERK1/2 (PD98059, 20 µM) or JNK (SP600125, 20 µM) for 30 min and then exposed to LPS (1 µg/ml) for 24 h. Secretion of TNF-α in culture supernatants was detected using ELISA. The results are expressed as the means ± SEMs of three independent experiments. *P<0.05 compared to the control.

The ability of LPS to stimulate the release of TNF-α was studied in BMMs using ELISA. Figure 1A shows that the production of TNF-α was dose dependently up-regulated in BMMs after 24 h of LPS stimulation. Increases were maximal at a concentration of 1 µg/ml. The MAPK pathway is known to play a substantial role in LPS-induced cytokine production in macrophages [14], [15]. To determine MAPK activation, the phosphorylation of p38 MAPK, ERK1/2, and JNK in BMMs with or without LPS stimulation were evaluated with western blotting. Our results revealed that, after stimulation with 1 µg/ml LPS, the phosphorylation of ERK1/2, p38 MAPK, and JNK in BMMs were significantly increased within 15 min (Figure 1B). To determine which of these kinases was relevant to the enhanced LPS-induced TNF-α production in BMMs, inhibitor studies were performed. Cells were cultured with MAPK inhibitor for 1 h before stimulation with 1 µg/ml LPS for 24 h. As shown in Figure 1C, SB203580, a specific inhibitor of p38 MAPK, significantly suppressed the LPS-induced production of TNF-α in BMMs as assessed by ELISA. However, the ERK1/2 inhibitor PD90859 and the JNK inhibitor SP600125 did not alter LPS-induced TNF-α production in BMMs.

### E_2_ down-regulates LPS-induced TNF-α production in BMMs

**Figure 2:**
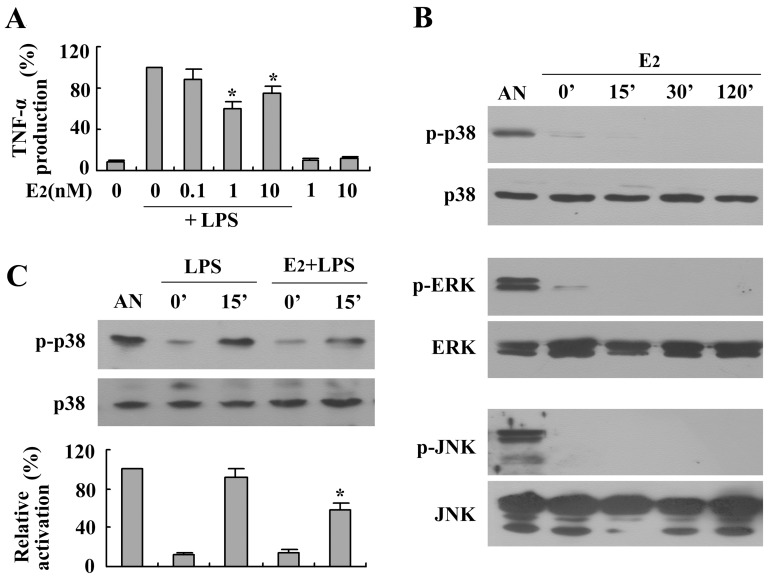
Effects of 17β-estradiol (E_2_) on LPS-induced TNF-α production and activation of MAPKs in BMMs. (A) Cells were treated with LPS (1 µg/ml) alone or in combination with different concentrations of E_2_ for 24 h, and the culture media were collected to measure TNF-α concentrations using ELISA. The relative expression of TNF-α was evaluated with the results obtained from LPS-stimulated macrophages. The results are expressed as the means ± the SEMs of three independent experiments. (B) Cells were treated with E_2_ (1 nM) for different periods. Protein extracts were subjected to western blotting to detect the phosphorylated and total forms of three MAPK molecules, p38 MAPK, ERK1/2 and JNK. Stimulation of the cells with anisomycin (AN, 10 µg/ml, 30 min) was used as a positive control. (C) Cells were stimulated with LPS (1 µg/ml) alone or in combination with E_2_ (1 nM) for 15 min. Protein extracts were subjected to western blotting to detect the phosphorylated and total forms of p38 MAPK. The relative activation of p38 was densitometrically evaluated. Representative blots are shown, and the results were verified by at least three independent experiments. *P<0.05 compared to the control and LPS.

To examine the effects E_2_ of on LPS-induced TNF-α production in BMMs, cells were incubated with different concentrations of E_2_ for 24 h, with or without LPS costimulation (1 µg/ml). As shown in Figure 2A, E_2_ significantly inhibited LPS-induced TNF-α production, and the maximum effect occurred at a physiological concentration of 1 nM. E_2_ by itself did not affect TNF-α production. We subsequently evaluated the effects of E_2_ on the LPS-induced phosphorylation of p38 MAPK, ERK1/2, and JNK. As shown in Figure 2B, E_2_ was not able to activate any of the three MAPK families at different time points over 2 h as detected by western blotting using specific anti-MAPK antibodies. However, costimulation with 1 nM E_2_ dramatically reduced the LPS-induced phosphorylation of p38 MAPK without altering total protein expression (Figure 2C). In contrast, E_2_ did not affect the LPS-induced activation of ERK1/2 and JNK (data not shown).

### Expression of iER in BMMs

**Figure 3:**
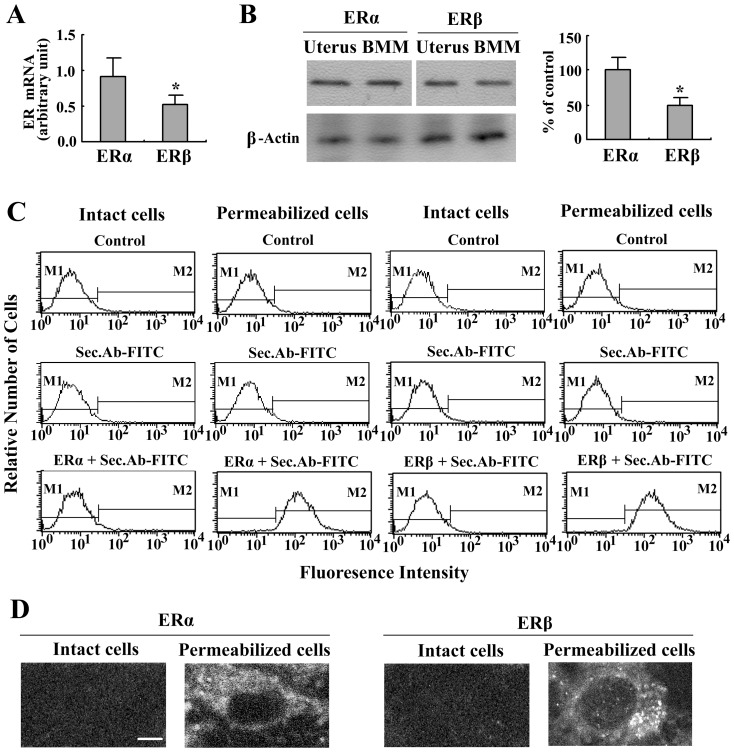
Detection of intracellular estrogen receptors in BMMs. (A) Real-time PCR analyses of ERα and ERβ mRNA from BMMs. The expression levels of these receptors are given as arbitrary units normalized to 18S rRNA expression. (B) Western blotting analyses of ERα and ERβ proteins from BMMs and mouse uterus. The relative expression of ER was densitometrically evaluated. (C) Flow cytometry of intact and permeabilized cells labeled with anti-ERα or anti-ERβ antibody followed by FITC-conjugated secondary antibody or by FITC-conjugated secondary antibody only. (D) Confocal laser scanning microscopy of intact and permeabilized cells labeled with anti-ERα or anti-ERβ antibody followed by FITC-conjugated secondary antibody. The results were verified by at least three independent experiments. The bar indicates 10 µm. *P<0.05 compared to the ERα.

Most, if not all, of the effects of estrogen are mediated by two members of the nuclear receptor superfamily: ERα and ERβ [5], [6]. The expression of intracellular ERα and ERβ in BMMs was detected. Using real-time PCR, we demonstrated that ERα mRNA was expressed at higher levels than ERβ in BMMs (Figure 3A). Consistent with these findings, total ERα protein levels were higher in BMMs as determined by western blotting (Figure 3B). Moreover, incubation of BMMs with the same ER antibody and FITC-conjugated secondary antibody produced significant labeling of permeabilized cells as detected by flow cytometry (Figure 3C). The majority of ER was localized in the cytoplasm of the BMMs, whereas the nuclei remained unlabeled as revealed by CLSM (Figure 3D).

### Expression of mER in BMMs

**Figure 4:**
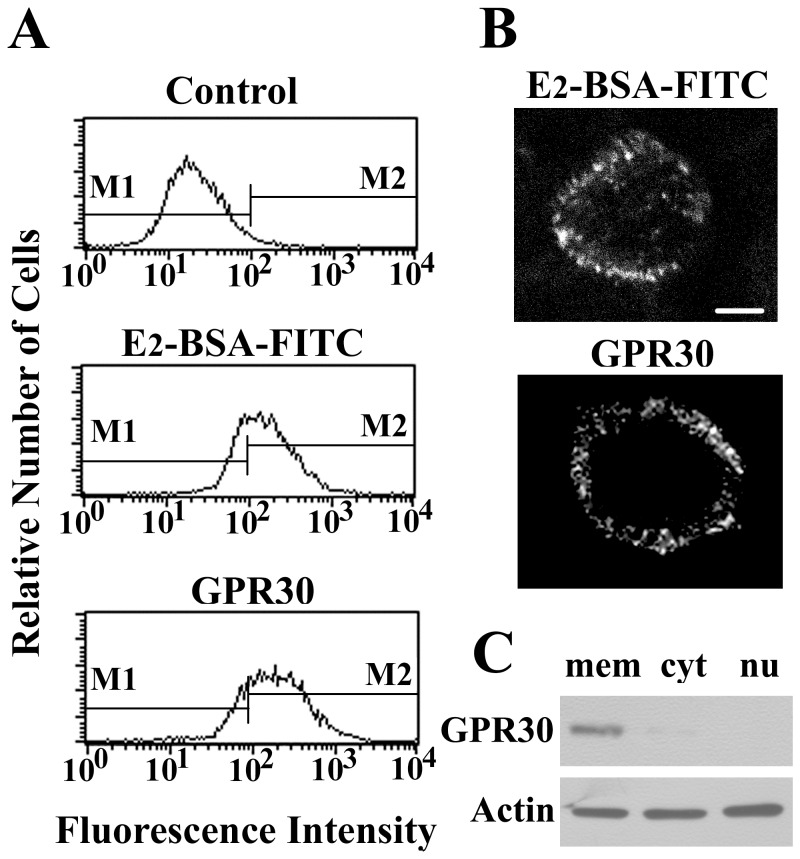
Detection of membrane estrogen receptors in BMMs. (A) Flow cytometry and (B) confocal laser scanning microscopy analyses of membrane estrogen receptors in BMMs. Intact cells labeled with E_2_-BSA-FITC or BSA-FITC alone as a control. For GPR30 detection, intact cells were pretreated with anti-GPR30 followed by FITC-conjugated secondary antibody. (C) Western blotting analyses of GPR30 protein from the membrane (mem), cytoplasmic (cyt), or nuclear (nu) fractions of BMMs. The results were verified by at least three independent experiments. The bar indicates 10 µm.

Estrogen also acts on the plasma membrane to initiate rapid signaling pathways in the cytoplasm and regulate cellular functions, and these mechanisms are referred to as the nongenomic pathway [21]–[24]. Here, using the cell-impermeable E_2_-BSA-FITC, we examined whether there was any estrogen binding at the surface of BMMs. After incubation with E_2_-BSA-FITC for 30 min, the cells exhibited a significant increase in fluorescence intensity compared to the controls as determined by flow cytometry (Figure 4A). Control treatment with BSA-FITC did not produce any binding activity, which suggests the membrane binding we observed was due specifically to E_2_ and not BSA. Furthermore, CLSM revealed specific membrane staining for E_2_-BSA-FITC, and the outline of a single cell is shown in Figure 4B. The expression of GPR30 on the surfaces of intact BMMs was also evaluated by flow cytometry (Figure 4A) and CLSM (Figure 4B), and the fluorescence pattern was identical to that observed with E_2_-BSA-FITC incubation. Consistent with these findings, western blotting analysis demonstrated that the GPR30 protein was retained within plasma membrane fraction (Figure 4C).

**Figure 5:**
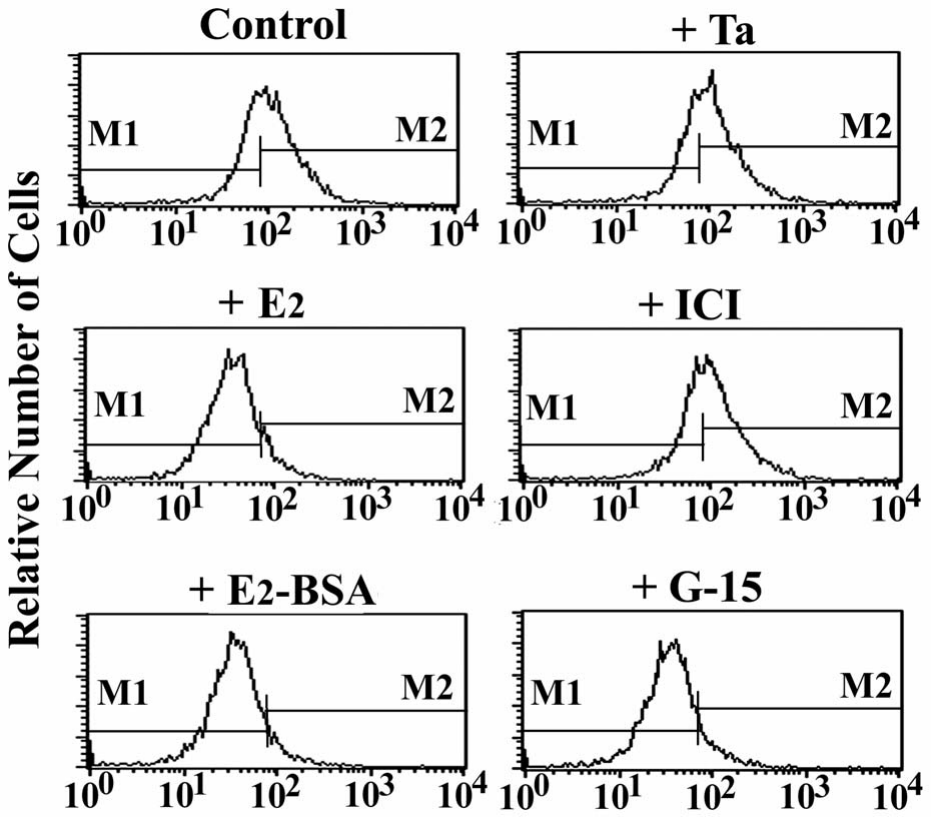
Specificity of E_2_-BSA-FITC binding on BMMs. Cells were incubated for 15_2_-BSA-FITC (1×10^−6^ M) in the absence (control) or in the presence of a 10-fold excess of different unlabeled materials: 17β-estradiol (E_2_), E_2_-BSA, tamoxifen (Ta), ICI 182780 (ICI), or G-15. Fluorescence intensity was analyzed by flow cytometry. The results were verified by at least three independent experiments.

To establish the specificity of E_2_-BSA-FITC binding, BMMs were incubated for 15 min with E_2_-BSA-FITC in the presence or absence of 10-fold excess of various agents, and fluorescence intensities were subsequently analyzed by flow cytometry. As shown in Figure 5, the binding of E_2_-BSA-FITC were competitively attenuated by E_2_ and E_2_-BSA. This membrane binding site for estrogen is not related to the classical iER because the iER inhibitors tamoxifen and ICI 182780 did not block of E_2_-BSA-FITC binding. Furthermore, the specific GPR30 inhibitor G-15 was an effective competitor and significantly reduced the fluorescence intensity of surface-bound E_2_-BSA-FITC.

### 17β-estradiol induced Ca^2+^ signaling via the mERs of BMMs

**Figure 6:**
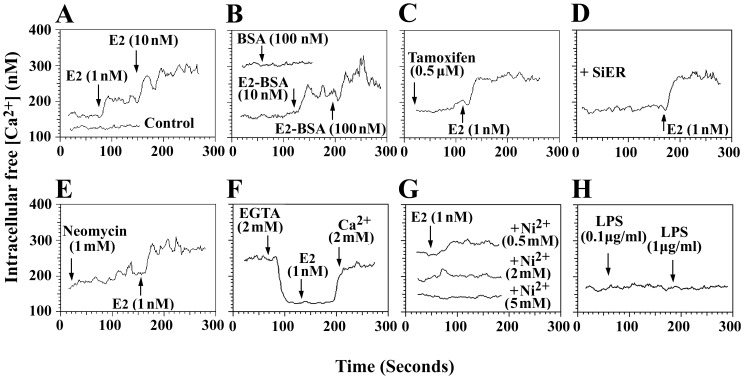
Effects of various agents on intracellular free Ca^2+^ concentration ([Ca^2+^]_i_) in BMMs. (A) Effects of 17β-estradiol (E_2_) and vehicle on [Ca^2+^]_i_. (B) Effects of E_2_-BSA and BSA alone (as a control) on [Ca^2+^]_i_. (C) Cells were pretreated with tamoxifen for 30 min before adding E_2_. (D) Effects of E_2_ on [Ca^2+^]_i_ in SiER-transfected BMMs. (E) Cells were preincubated with neomycin for 5 min before adding E_2_. (F) Cells were preincubated with EGTA for 1 min before adding E_2_. (G) Cells were preincubated with different doses of Ni^2+^ for 5 min before adding E_2_. (H) Effects of LPS on [Ca^2+^]_i_. The results were verified by at least three independent experiments. Arrows in each curve indicate the addition of substances to the BMMs suspensions.

Estrogens have been shown to induce the rapid activation of kinase-signalling cascades and to modulate intracellular Ca^2+^ levels. These effects are considered to be nongenomic because they are too rapid to involve changes in gene transcription [24]. The effects of E_2_ on the intracellular Ca^2+^ levels of BMMs were investigated using Fura-2/acetoxymethylester and fluorescence spetrophotometry. As shown in Figure 6A, E_2_ induced a rapid and sustained increase in [Ca^2+^]_i_ in the BMMs. To evaluate whether this effect of estrogen was mediated by extracellular membrane receptors, we tested the effect of E_2_-BSA on BMMs. Figure 6B shows that E_2_-BSA induced intracellular Ca^2+^ increases and that this response was similar to that obtained with free E_2_. BSA alone did not produce any change in [Ca^2+^]_i_. If iERs are responsible for the E_2_-induced Ca^2+^ increase in BMMs, this increase should be blocked by tamoxifen, which is an antagonist of iER. However, Ca^2+^ elevations were not affected by 30 min of preincubation of the cells with tamoxifen (Figure 6C). Moreover, knock down of the iERs using siRNA did not block the E_2_-induced Ca^2+^ increase (Figure 6D). The increase in [Ca^2+^]_i_ was the result of either extracellular Ca^2+^ influx through the plasma membrane or intracellular Ca^2+^ release from endoplasmic reticulum. Thus, we blocked the release of Ca^2+^ from intracellular stores with neomycin, a phospholipase C inhibitor, and this treatment did not prevent the E_2_-induced rise in [Ca^2+^]_i_ (Figure 6E). When extracellular Ca^2+^ was removed by adding EGTA before E_2_, the E_2_-induced [Ca^2+^]_i_ elevation was completely abolished (Figure 6F). The above results indicate that the E_2_-induced increase in [Ca^2+^]_i_ was due to an influx of extracellular Ca^2+^. This influx was channel-mediated because increasing doses of the Ca^2+^ channel blocker Ni^2+^ caused gradual decreases in E_2_-induced Ca^2+^ influxes, and 5 mM Ni^2+^ completely blocked the influx of Ca^2+^ (Figure 6G). LPS stimulation did not induce calcium influx in BMMs. (Figure 6H).

### E_2_ down-regulates LPS-stimulated TNF-α production through a nongenomic signaling pathway in BMMs

**Figure 7:**
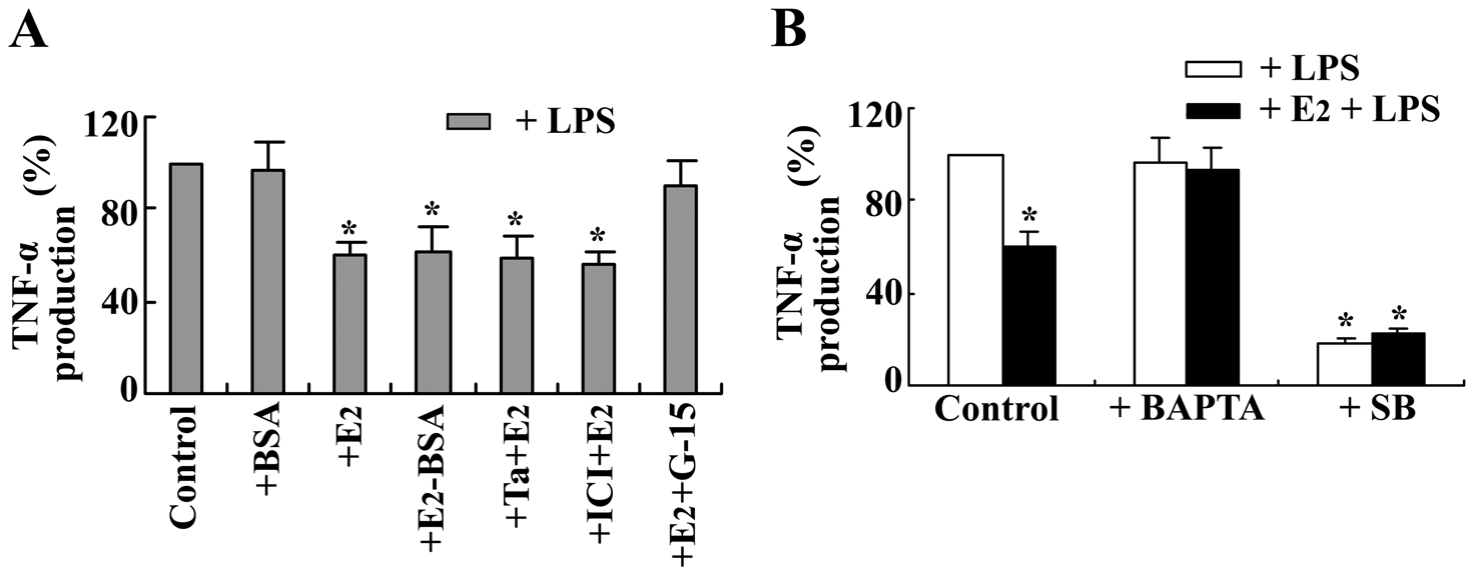
Effects of various agents on estrogenic control of LPS-induced TNF-α production in BMMs. (A) Cells were incubated for 24 h with 1 µg/ml LPS alone or in the presence of BSA (10 nM), 17β-estradiol (E_2_, 1 nM), E_2_-BSA (10 nM), E_2_ plus a 10-fold excess of tamoxifen (Ta), ICI 182780 (ICI), or G-15. (B) Cells were preincubated with BAPTA (10 µM) or SB203580 (SB, 20 µM) for 30 min before the addition of LPS (1 µg/ml) or LPS plus E_2_ (1 nM) for 24 h. The culture media were collected to measure TNF-α concentrations using ELISA. The relative expression of TNF-α was evaluated with results obtained from LPS-stimulated macrophages. The results are expressed as the means ± the SEMs from three independent experiments. *P<0.05 compared to the LPS control.

To further explore the mechanism of the inhibitory effect of E_2_ on LPS-induced TNF-α production in BMMs, cells were pretreated with the iER antagonist tamoxifen or ICI 182780 for 30 min before the addition of E_2_ and LPS. As shown in Figure 7A, E_2_ attenuated LPS-induced TNF-α production, and this inhibitory effect was not antagonized by tamoxifen or ICI 182780. These data indicate that E_2_ attenuates LPS-induced TNF-α secretion in an iER-independent manner in BMMs. In contrast, the impermeable E_2_-BSA exerted an inhibitory effect on LPS-induced TNF-α secretion that was similar to that of E_2_, and BSA did not produce any change in LPS-induced TNF-α production. Furthermore, the specific GPR30 antagonist G-15 significantly blocked the effect of estradiol on LPS-induced TNF-α production. These data suggest that the effect of E_2_ in LPS-induced TNF-α production is mediated through mERs.

**Figure 8:**
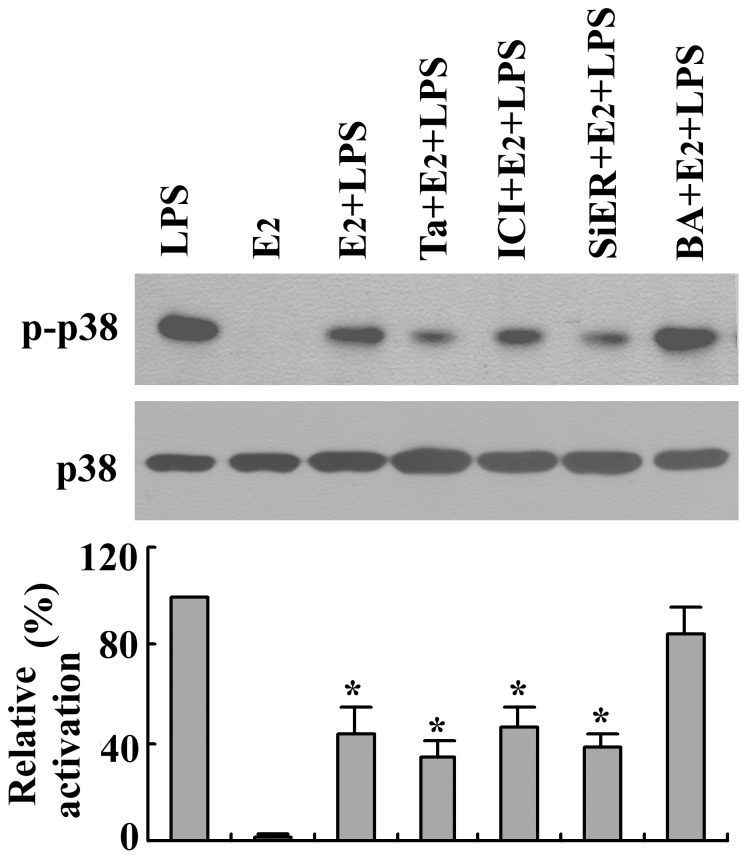
Effects of various agents on estrogenic control of LPS-induced p38 activation in BMMs. The cells were stimulated by LPS (1 µg/ml) for 15 min alone or combined with 17β-estradiol (E_2_, 1 nM), E_2_ plus tamoxifen (Ta, 10 nM), E_2_ plus ICI 182780 (ICI, 10 nM), or E_2_ plus BAPTA (BA, 10 µM). Cells were preincubated with tamoxifen, ICI 182780, or BAPTA for 15 min before the addition of LPS. On lane 5, cells were transfected with siRNA of iER for 72 h before the LPS stimulation. Protein extracts were subjected to western blotting to detect the phosphorylated and total forms of p38 MAPK. The relative activation of p38 was densitometrically evaluated. Representative blots are shown, and the results were verified by at least three independent experiments. *P*<0.05 compared to LPS and E_2_.

When BMMs were preincubated with intracellular Ca^2+^ chelator BAPTA, the inhibitory effect of E_2_ on LPS-induced TNF-α production was abrogated, which suggests that Ca^2+^ is involved in this effect of E_2_ (Figure 7B). We know that the effect of estradiol in LPS-stimulated TNF-α production in BMMs is mediated through p38 MAPK. In the present study, when LPS-induced TNF-α production was abolished by SB 203580, the remaining TNF-α production was no longer regulated by E_2_ (Figure 7B). Moreover, the inhibitory effect of E_2_ on LPS-induced p38 MAPK phosphorylation was abrogated by BAPTA but not by an iER antagonist or siRNA as demonstrated in western blotting analyses (Figure 8).

## Discussion

E_2_ is the major circulating estrogen in pre-menopausal females, and it has a substantial role in the modulation of innate immune function [2], [3]. Understanding the effects of estrogens on macrophage function will provide important insights into the mechanisms by which these sexual steroid hormones affect immune and inflammatory responses in women. Numerous studies have shown that E_2_ regulates production of proinflammatory cytokines by macrophages [4], [28], [29]. In the present study, we examined the effects of E_2_ on TNF-α production following LPS stimulation and explored the related mechanisms in BMMs.

We found that the potent macrophage activator LPS increased TNF-α production in BMMs. E_2_ itself did not affect TNF-α production, but it inhibited LPS-inducible TNF-α production in BMMs. Moreover, E_2_ selectively attenuated the LPS-induced activation of p38 MAPK, but not ERK1/2 and JNK phosphorylation. The blocking of the LPS-induced phosphorylation of p38 MAPK by E_2_ might responsible for its inhibitory effect on LPS-induced TNF-α production. The mechanisms by which E_2_ regulated LPS-induced phosphorylation of p38 MAPK in BMMs were further explored. Two major pathways, generally termed genomic and nongenomic, are known to mediate the effects of E_2_ on cells [20], [21]. It was possible that E_2_ inhibited p38 MAPK through a genomic pathway mediated by the classical iER and/or through an iER-independent nongenomic pathway.

According to the classical hypothesis, the cellular effects of estrogens are mediated by iERs, which serve as transcription factors. iERs are expressed in two forms: ERα and ERβ [5], [6]. Although BMMs were shown to express both ERα and ERβ, the inhibitory action of E_2_ on LPS-stimulated TNF-α production was not sensitive to the iER blockers tamoxifen and ICI 182780, which excluded the possibility that the action of E_2_ on LPS-induced TNF-α expression is mediated by iER-mediated genomic pathways in BMMs.

Nongenomic actions are initiated at the plasma membrane and are postulated to be mediated by mERs. E_2_-BSA has been shown to be a plasma membrane impermeable compound and has been used to study the role of surface estrogen receptors in producing nongenomic effects on cellular functions [30]. The present study provided evidence for the presence of mERs on the surface of BMMs. Flow cytometry and CLSM revealed the binding of E_2_-BSA-FITC on the plasma membranes of intact BMMs. Some authors have stated that mERs are largely identical or structurally related to intracellular ERα or ERβ in various cells [31]–[33]. To test this possibility, intact BMMs were incubated with anti-ERα or anti-ERβ antibody. Neither antibody produced any significant fluorescence on intact BMMs as examined by flow cytometry. Furthermore, the iER blockers tamoxifen and ICI 182780 were not able to inhibit the binding of E_2_-BSA-FITC to BMMs. In contrast, CLSM revealed that the classic iERs in BMMs were not accessible from the outer surface of intact cells but could only be detected intracellularly. Therefore, it is likely that E_2_-BSA does not act via membrane receptors that are related to iERs on BMMs. It has been reported that GPR30 may function as a novel transmembrane estrogen receptor and can mediate rapid nongenomic events [25]. The expression of GPR30 on the surface of intact BMMs was evaluated by flow cytometry and CLSM, and the fluorescence patterns were identical to that observed after E_2_-BSA-FITC incubation. Consistent with these findings, western blotting analysis demonstrated that the GPR30 protein is retained within the plasma membrane fraction. Moreover, the membrane binding of E_2_-BSA-FITC was competitively inhibited by the specific GPR30 inhibitor G-15. These data suggest that the nongenomic actions of E_2_ are mediated via GPR30 on membranes of BMMs.

Nongenomic actions manifest themselves as rapid responses of target cells that range from seconds to minutes [24], [34]. Here, nongenomic E_2_ signaling manifested itself as a rapid rise in [Ca^2+^]_i_ in the BMMs that occurred within seconds. Moreover, we excluded the possibility that E_2_ acted through iERs because neither tamoxifen or iER silence affected the rapid Ca^2+^ increase induced by E_2_. This E_2_-induced increase of [Ca^2+^]_i_ might be initiated on the cell surface via specific E_2_-receptors. Indeed, the binding of the plasma membrane impermeable E_2_-BSA also induced a rapid increase in [Ca^2+^]_i_, which supports the notion that the surface estradiol receptors on BMMs are functionally coupled to intracellular Ca^2+^ homeostasis. mERs could mediate the E_2_-induced rise in [Ca^2+^]_i_ possibly via the influx of extracellular Ca^2+^ and/or Ca^2+^ release from intracellular stores in BMMs. When extracellular Ca^2+^ was removed with EGTA, the E_2_-induced [Ca^2+^]_i_ elevation was totally abolished. Moreover, our data showed that blocking the release of Ca^2+^ from intracellular stores with neomycin, a phospholipase C inhibitor that binds to phosphoinositides, did not prevent the E_2_-induced rise in [Ca^2+^]_i_. These data suggest that the effects of estradiol are not due to the release of Ca^2+^ from intracellular Ca^2+^ stores but are rather due to an influx of extracellular Ca^2+^. In BMMs, the external Ca^2+^ influx was not due to diffusion but was channel-mediated because the specific Ca^2+^ channel blocker Ni^2+^ completely blocked the E_2_-induced Ca^2+^ influx.

MAPK pathways have been reported to be involved in the nongenomic E_2_ cascade in various types of cells [35], [36]. In the present study, E_2_ itself did not affect TNF-α production in BMMs. Interestingly, pretreatment of the cells with E_2_ attenuated the LPS-induced activation of p38 MAPK and subsequent production of TNF-α. These E_2_ effects were not mediated through iERs; rather, they were mediated through nongenomic signaling that manifested itself as an E_2_-induced rapid rise in [Ca^2+^]_i_ in BMMs. This view is supported by the following findings. First, the inhibitory effect of E_2_ on LPS-induced TNF-α production could not be prevented with iER inhibitors such as ICI 182780 and tamoxifen. Second, the impermeable E_2_-BSA was capable of producing rapid rises in [Ca^2+^]_i_ and also exerted a significant inhibitory effect on LPS-induced TNF-α secretion that was similar to the effect of E_2_. Third, when BMMs were preincubated with the intracellular Ca^2+^ chelator BAPTA, E_2_ lost its effect on LPS-induced p38 MAPK phosphorylation. Indeed, BAPTA also abolished the inhibitory effect of E_2_ on LPS-stimulated TNF-α secretion by blocking intracellular free Ca^2+^ accumulation. Therefore, E_2_ exerted its inhibitory effect on LPS-stimulated TNF-α production through nongenomic Ca^2+^ signaling in BMMs.

In summary, our present study revealed the presence of functional ERs on the membranes of BMMs that did not mediate the classical genomic response; rather, these receptors initiated a novel nongenomic estradiol signaling pathway that involved Ca^2+^ as an intracellular mediator. This E_2_-induced nongenomic Ca^2+^ signaling through mERs that was independent of the classical nuclear ER was responsible for the inhibitory effect of E_2_ in LPS-induced TNF-α production. As a second messenger, intracellular Ca^2+^ is essential for many cellular responses. This inhibitory effect of E_2_ on LPS signaling of macrophages that is mediated by nongenomic Ca^2+^ signaling will possibly advance the exploitation of the effects of estrogen for several human inflammatory diseases.
